# Surface topography scan as a source for orthotic brace design. Technique and case presentation

**DOI:** 10.1186/1748-7161-9-S1-O57

**Published:** 2014-12-04

**Authors:** Krzysztof Mularczyk, Wojciech Glinkowski, Robert Sitnik

**Affiliations:** 1 Institute of Micromechanics and Photonics, Warsaw University of Technology, Warsaw, Poland; 2 Chair and Department of Orthopaedics and Traumatology of Locomotor System, Baby Jesus Clinical Hospital, Medical University of Warsaw, Warsaw, Poland; 3 Institute of Micromechanics and Photonics, Warsaw University of Technology, Warsaw, Poland

## 

Accurate adjustment and individualized brace design may warrant the optimal brace manufacturing that fit the patient's body surface. Computerized methods may improve bracing treatment.

## Material and methods

Computer Aided Design and Manufacturing in bracing is based on the introduction of appropriate data to the computer using anthropometry. The best data can be delivered by precise 3D surface topography supplemented with X-ray and other necessary radiographic modalities. The surface topography scanning used for this study is based on structured light projection. Scanning the surface of the patient's body is carried out using the multi-directional laboratory scanning system, located at the Baby Jesus Clinical Hospital. The scanning system consists of four uni-directional systems. Each system is calibrated in a common coordinate system and a PC computing unit. Each unit includes a digital camera, multimedia projector and independent PC operating for the projection and recording of images (raster images and binary sinusoidal modulation of brightness). Raster images are recorded by the camera and processed by the scanner's software. It allows extracting the 3D shape of the patient’s image of the torso surface. The total time of image acquisition is approximately 1.5 seconds. It can be further modified by the orthotist. The positive, foam torso model can be manufactured upon approval and acceptance.

The measurement data are obtained in the form of a point cloud further converted into a 3D model in STL format for further manufacture the orthosis. Point cloud accuracy is as high as less than 0.5 mm. The methodology is described based on the real AIS case.

## Results and conclusion

The case of 12 years old AIS female patient was documented while elaboration the 3D scan and manufacturing of the brace based on STL file derived from 3D point cloud. Presented approach shows the implementation of the 3D surface topography scanning and its opportunity to transform it easy into a brace design suitable for TLSO brace manufacturing. Good fit and function today remains an important issue in brace manufacturing. We believe that 3D 360 degrees surface scan may significantly improve the accuracy of individual brace production.

## Consent

Written informed consent was obtained from the parents/legal guardian of the patient for publication of this Case report. A copy of the written consent is available for review by the Editor of this journal.

